# Sensitization against skin resident fungi is associated with atopy in cholinergic urticaria patients

**DOI:** 10.1186/s13601-020-00324-z

**Published:** 2020-06-01

**Authors:** Sabine Altrichter, Pia Schumacher, Ola Alraboni, Yiyu Wang, Makiko Hiragun, Michihiro Hide, Marcus Maurer

**Affiliations:** 1 Department of Dermatology and Allergy, Allergie-Centrum-Charité/ECARF, Charité-Universitätsmedizin Berlin, Charitéplatz 1, 10117 Berlin, Germany; 2 Department of Dermatology, Integrated Health Sciences, Institute of Biomedical and Health Sciences, Hiroshima University, Hiroshima, Japan

**Keywords:** Cholinergic urticaria, IgE, Sensitization, Skin fungi

## Abstract

**Background:**

Cholinergic urticaria (CholU) is a common type of chronic inducible urticaria, characterized by small itchy wheals that appear upon physical exercise or passive warming. *Malassezia globosa*, a skin resident fungus, has been identified as an antigen that induces mast cell/basophil degranulation and wheal formation through specific IgE, in Japanese patients with atopic dermatitis and CholU. In this study we aimed in assessing the rate of IgE sensitizations against skin resident fungi in European CholU patients.

**Methods:**

We assessed serum IgE levels to *Malassezia furfur, Candida albicans* and *Trichophyton mentagrophytes* using routine lab testing and *Malassezia globosa* using a newly established ELISA. We correlated the results to wheal formation and other clinical features.

**Results:**

Four patients (of 30 tested) had elevated levels of IgE against *Malassezia furfur* and *Candida albicans* and two had elevated levels of IgE against *Trichophyton mentagrophytes.* Four sera (of 25 tested) had elevated levels of IgE to the *Malassezia globosa* antigen supMGL_1304. Sensitization to one skin fungus was highly correlated with sensitization to the other tested fungi. We saw highly significant correlations of sensitization to supMGL_1304 with wheal size in the autologous sweat skin test (r_s_ = 0.7, *P* = 0.002, n = 19), the Erlangen atopy score (r_s_ = 0.5, *P* = 0.03, n = 19), total IgE serum levels (r_s_ = 0.5, *P* = 0.04, n = 19) and a positive screen for IgE against common airborne/inhalant allergens s (sx1; r_s_ = 0.54, *P* = 0.02, n = 19).

**Conclusions:**

Sensitization to skin resident fungi including *Malassezia globosa* is uncommon in European CholU patients, but is associated with atopy and pronounced wheal formation upon dermal contact with their own sweat.

*Trial registration* German Clinical Trials Registry DRKS-ID: DRKS00004277

## Background

Cholinergic urticaria (CholU) is one of the most common forms of inducible urticaria and is characterized by small itchy wheals that are induced by physical activity or passive warming [1–3]. There is a high reported prevalence (up to 4%) in the general population [4, 5], with one publication mentioning a 20% prevalence in young adults [6], although the disease can also start later in life [7]. The severity/clinical spectrum of the disease might be broad, with some patients suffering from severe disease with frequent wheal formation and intolerable itch, which majorly reduces their quality of life [8, 9].

The underlying pathogenesis of CholU is not understood, but several mechanisms have been proposed [10, 11]. Many studies have provided evidence that IgE-mediated mast cell activation is of major importance in the pathogenesis of CholU. Patients exhibit high rates of atopy [12], which has been linked to higher levels of disease activity and to elevated total IgE serum levels. CholU is adoptively transferable to healthy subjects by injecting CholU patient serum into healthy skin, suggesting a transferable serum factor and antigen–antibody driven disease mechanism [13]. It is also well established that some CholU patients develop wheal and flare type reactions upon skin testing with their own sweat [13], pointing towards a type-I allergy against sweat. Further evidence that IgE-mediated effects play a major role in CholU comes from reports of successful treatment of patients with anti-IgE (omalizumab) [14, 15]. Nevertheless, the antigen causing the IgE-mediated symptoms in CholU was not known for a long time. More recently, Hiragun et al. detected elevated levels of IgE against the lipophilic yeast-like fungi *Malassezia globosa* in patients with atopic dermatitis and CholU and identified a frequently reactive antigen (supMGL_1304) in human sweat [16–18]. Sweat-derived antigens have also been shown to be of functional relevance, as these antigens were able to induce degranulation of mast cells and basophils in sensitized atopic dermatitis patients [19, 20], a disease where skin resident fungi are thought to play an important role in the pathogenesis of the disease [21].

These studies were performed in Japan. To date it is not known whether patients from other parts of the world also show these sensitizations, whether they account for the skin reaction with autologous sweat and whether they are of clinical relevance. To address this, we analyzed specific IgE serum levels to known skin resident fungi, including the *Malassezia globosa* antigen supMGL_1304, and correlated the results with wheal formation in the autologous sweat skin test (ASwST) and other clinical features.

## Materials and methods

### Study subjects

In this study, 39 patients with CholU were recruited at the UCARE Center [22] of the Department of Dermatology and Allergy, Charité-Universitätsmedizin, Berlin for provocation testing, sweat collection, skin testing and extensive clinical characterization. Patients were advised to stop taking antihistamines at least 3 days prior to any tests. None of the patients had taken local or systemic glucocorticoids or other immunosuppressive therapy 2 weeks before the tests. As a control group, 70 healthy individuals were recruited and underwent the same procedures, except provocation testing. The study was approved by the Ethics Committee of the Charité-Universitätsmedizin Berlin (EA4/124/10), and is registered in the German Clinical Trials Registry (DRKS-ID: DRKS00004277). Other aspects of the study will be published elsewhere.

Demographic and clinical characteristics of the patients and healthy controls are shown in Table 1.

**Table 1:** Clinical characteristics of study participants

	CholU patients n = 39	Healthy controls n = 70	*P* value
Age [years]			
Median (IQR)	31.5 (23.3–46.5)	31.0 (27.0–34.0)	n.s.
Sex			
M:F	16:23	40:30	n.s.
BMI [kg/m^2^]			
Median (IQR)	24.2 (20.8–26.7)	22.9 (20.2–24.7)	n.s.
Persistance of disease [years]			
Median (range)	6.0 (3.0–12.8)	–	–
Total IgE [kU/l]			
Median (range), n = 29	164.0 (46.8–291.0)	–	–

*IQR* interquartile range, *M* male, *F* female

### Clinical assessments

Patients and healthy controls were assessed for their clinical history and comorbidities. Atopic skin diathesis (atopic predisposition) was also assessed using the Erlanger Atopy score questionnaire [23]. For CholU patients, disease duration was recorded and disease severity was rated using the Cholinergic Urticaria Severity Index (CholUSI). This is a sum score that takes into account the frequency of CholU symptoms (< once a month = 0 point; once a month = 1 point; > once a month = 2 points; once a week = 3 points; > once a week = 4 points; daily = 5 points; > daily = 6 points), eliciting factors (one point each for: physical exercise, hot bath, hot shower, emotional stress, hot food, sauna, other), duration of skin lesions (< 5 min = 0 point; 5 – 10 min = 1 point; 10–20 min = 2 points; 20–30 min = 3 points; 30–60 min = 4 points; > 1 h = 5 points) and itch (none = 0 point; mild = 1 point; moderate = 2 points; severe = 3 points). The CholUSI score ranges from 0 to 21 points: < 5 points = very mild CholU; 5–9 points = mild CholU; 10–15 points = moderate CholU; > 15 points = severe CholU [24].

### Sauna provocation test

After showering without soap and completely drying, patients were put in a large plastic bag (polyethylene, food safe grade, Ratioform GmbH, Pliening, Germany) that covered the entire body, apart from the head. They then took a 15 min sauna at 80 °C, during which sweat was collected in the bag. The total sweat volume was measured and the sweat was immediately frozen at − 20 °C and at − 80 °C for long term storage.

### Pulse controlled ergometry test

Eighteen patients underwent pulse controlled ergometry testing as described elsewhere [25]. They were assessed for the time to onset of sweating using the iodine–starch reaction (sweat test according to Minor), the time to onset of whealing and the increase in heart rate at the onset of whealing. Patients were assessed for their symptoms at the end of the test (itching: no itch = 0, mild itch = 1, moderate itch = 2, severe itch = 3; whealing: no whealing = 0, 1–20 wheals = 1, 21–50 wheals = 2, > 50 wheals = 3) to result in a urticaria activity score at the time of provocation (UASprovo) with a sum score between 0 and 6 points.

### Skin tests

Skin tests were performed at least 24 h after provocation testing. For the ASwST, the patient’s sweat was defrosted on the day of testing, sterile filtered using Sartorius™ Minisart NML (sterile, Sartorius AG, Göttingen, Germany) and diluted using sterile NaCl 0.9% under sterile conditions. The sweat was diluted (1:100) and intracutaneously injected (i.c., 50 µl) using a 1 ml syringe (Plastipak™, 1 ml, Becton–Dickinson (BD), Heidelberg, Germany) and a needle for subcutaneous injection (27 G 1/2`, 13 mm, BD), as described by Kozani et al. [21].

For the autologous serum skin test (ASST) [26], 50 µl freshly drawn patient serum was injected intradermally. For the acetylcholine i.c. (ACh i.c.) test, acetylcholine was diluted with NaCl 0.9% solution under sterile conditions to a final concentration of 100 µg/ml (0.01%) as described by Fukunaga et al. [27] and injected intradermally. Detailed results of the ACh i.c. test will be reported elsewhere.

NaCl 0.9% solution and histamine 100 µg/ml (0.01%; Bencard Allergie GmbH, Munich, Germany) were used for i.c. testing as negative and positive controls, respectively. The diameter of the resulting wheal and flare reactions were measured using a transparent ruler with 1 mm grading after 15 min and 30 min. The skin tests were considered positive if the wheal induced by sweat or ACh was ≥ 1.5 mm bigger than that induced by the negative control (NaCl 0.9%).

### IgE measurements

IgE levels were measured using the Immuno CAP System^®^ (Phadia Laboratory Systems, Thermo Fisher Scientific Inc, Uppsala, Sweden) at a central laboratory (Labor Berlin GmbH, Berlin, Germany). Serum was analysed for total IgE, IgE against common airborne/inhalant allergens (D1 house dust mite, E1 cat, E5 dog, G6 timothy, G12 rye, M2 Cladosporium herbarum, T3 birch, W6 mugwort), and specific IgE to *Candida albicans,Malassezia furfur* and *Trichophyton mentagrophytes*.

Total IgE levels > 100 kU/l were considered elevated. Specific IgE levels of > 0.1 kU/l were considered as detectable, and levels of ≥ 0.35 kU/l as sensitization (0.00–0.34 CAP-Class 0, 0.35–0.70 CAP-Class 1, 0.70–3.50 CAP-Class 2, 3.50 to < 17.5 CAP Class 3, 17.5 to < 50.0 CAP Class 4, 50.0–100.0 CAP-Class 5, > 100.0 CAP-Class 6).

### SupMGL_1304 IgE ELISA

IgE against supMGL_1304 from *Malassezia globosa* were detected using an ELISA as described by Kan et al. and Hiragun et al. [17, 28]. In brief, 96-well plates (high-binding, Greiner bio-one, Frickenhausen, Germany) were coated with 10 µg/ml mouse monoclonal antibody against purified supMGL_1304 (Smith-2 antibody; generation of antibody has been described in [18] overnight at 4 °C. Plates were blocked with 2% bovine serum albumin in phosphate buffered saline (BSA-PBS) for 1 h at room temperature and washed with 0.05% tween20-PBS. The coated plates were then incubated with 1 μg/ml purified supMGL_1304 in 0.1% BSA-PBS for 90 min at room temperature. Patients’ serum was diluted 1:40 with 1% BSA-PBS and incubated after washing of the plates at room temperature for 90 min. After washing, the plates were incubated with peroxidase-labeled antibody to human IgE (ε-chain specific, KPL, Gaithersburg, MD, USA) diluted 1:3000 with 1% BSA-PBS at room temperature for 1 h. After washing, TMB Microwell Peroxidase Substrate was added and incubated for 30 min. The reactions were stopped with TMB Stop Solution (KPL). The optical density was read at 450 nm using an automatic plate reader (Benchmark Plus, Bio-Rad, Hercules, CA, USA). Pooled serum of Japanese patients with atopic dermatitis was used as standard in various dilutions (1:10 to 1.1208 in 1% BSA-PBS). Results are given as arbitrary units (AU) in correlation with the standard serum.

### Statistical analyses

Statistical analyses were performed using IBM SPSS Statistics version 23 and GraphPad Prism Version 6.0. Binominal variables were analyzed using Chi-square test or Fisher Exact test for small categorical numbers (< 5). Non-parametric continuous variables were compared using Mann–Whitney-U test. Correlations were analyzed using Spearman rank test. *P *< 0.05 was considered to indicate statistical significance.

## Results

### Sensitization against skin resident fungi can be detected in a small population of European CholU patients

**Fig. 1:**
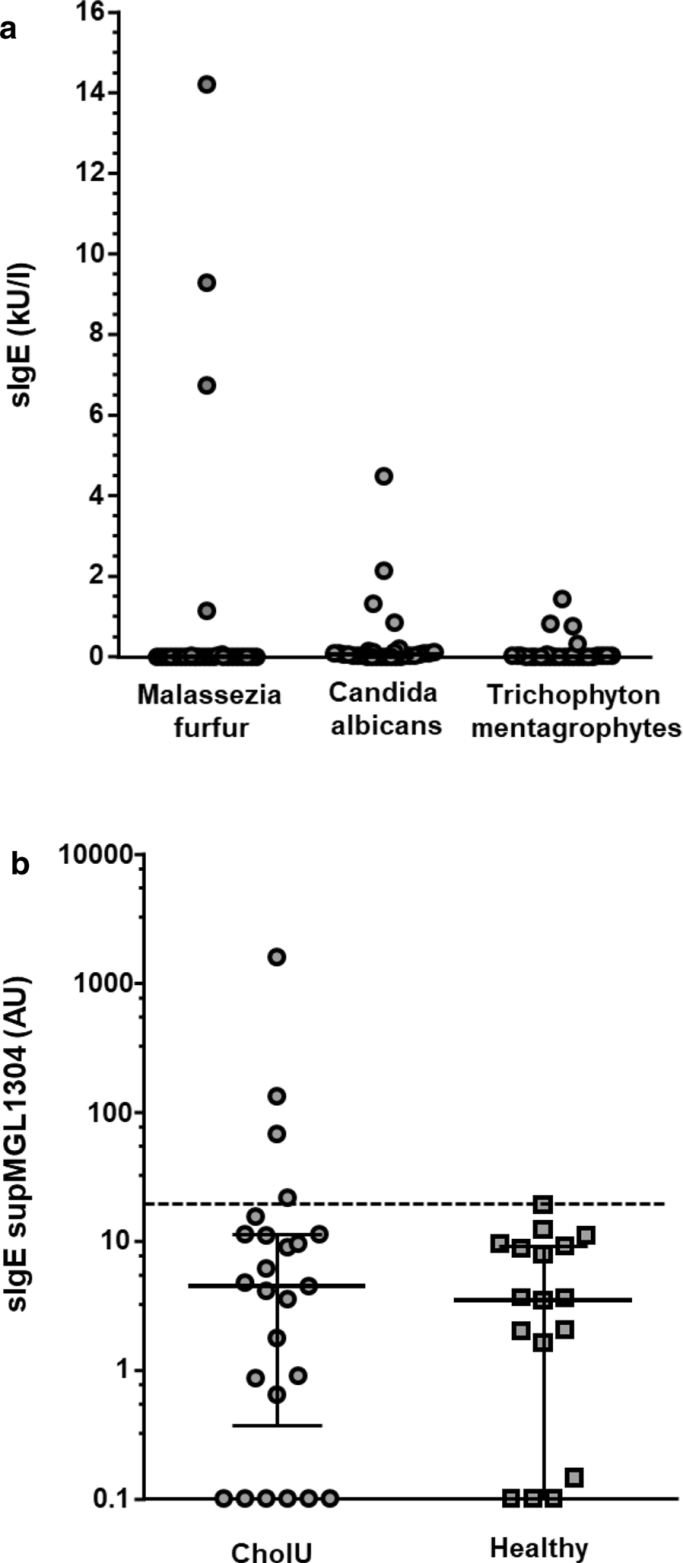
Specific IgE levels against skin fungi in CholU patients (n = 30) (**a**), and against supMGL-1304 in CholU patients (n = 25) and healthy controls (n = 20) (**b**). Individual patients are displayed as dots. **b** Shows median and interquartile ranges of specific IgE levels against supMGL-1304. 95% percentile calculated from healthy controls is represented by a dashed line

Results from the specific IgE testing against the fungi *Candida albicans a*, *Malassezia furfur* and *Trichophyton mentagrophytes* were available for 30 patients (Fig. 1a). Considering 0.1 kU/l as the limit for specific IgE levels, four patients (13%) had clearly elevated levels against *Malassezia furfur*. Two patients had IgE levels in the CAP-Class 2, and two had IgE levels in the CAP-Class 3, reaching levels up to 14.2 kU/l. More patients (n = 8; 26%) had detectable IgE levels against *Candida albicans,* but half of them were in the CAP-Class 0. Three had levels in CAP-Class 2 and one in CAP-Class 3. IgE against *Trichophyton mentagrophytes*. was detected in only three patients (10%), with one patient in CAP-Class 0 and two in CAP-Class 2.

Specific IgE against *Malassezia furfur* positivity was highly correlated with detectable levels of IgE against the other fungi (*Candida albicans.*: r_s_ = 0.59, *P* = 0.001; *Trichophyton mentagrophytes*: r_s_ = 0.68, *P* < 0.001).

### IgE against the *Malassezia globosa* antigen supMGL_1304 was elevated in a small number of CholU patients and was well correlated with levels of specific IgE against the other skin fungi

Using IgE levels from 20 healthy controls, who reported no known sensitization and had a normal low total IgE level below 100 kU/l, a cut-off was set at the 95% percentile (19.4 AU).

Of the 25 CholU patients who underwent testing, four (12%) had levels of IgE against supMGL_1304 above the set 95% percentile, with values ranging from 21.8 to 1594.5 AU (Fig. 1b). These values showed a high correlation with sensitization against the measured skin resident fungi (*Malassezia furfur* r_s_ = 0.633, *P* = 0.013; *Candida albicans*: r_s_ = 0.548, *P* = 0.037; *Trichophyton mentagrophytes*: r_s_ = 0.555, *P* = 0.042). Three of the four positive sera also showed elevated IgE levels to all the other three skin resident fungi.

### Sensitization to supMGL_1304 was correlated with skin reactions against autologous sweat in CholU patients, but not with ASST or ACh i.c. positivity

**Fig. 2:**
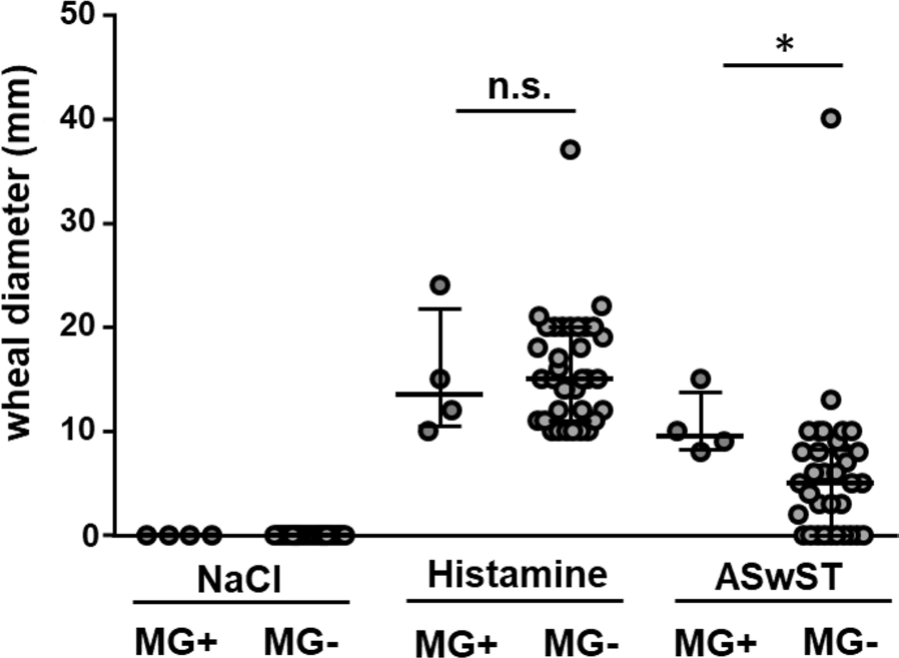
Skin reactivity in the ASwST with histamine as positive and NaCl as negative controls. Differences between the CholU patients with elevated specific IgE levels against supMGL-1304 (MG+; n = 4) and CholU patients with normal low supMGL-1304 (MG−, n = 21) are shown. Median and interquartile ranges of the largest wheal size measured are depicted

The ASwST (using 1:100 diluted sweat) was performed in 38 CholU patients. Twenty-eight patients (73.7%) developed a wheal 15 min after injection, compared with only one (1.4%) of the 69 healthy controls (*P *< 0.001). All four CholU patients with sensitization to supMGL_1304 had a positive reaction in the ASwST. Of the 34 patients without sensitization to supMGL_1304, 24 (73.5%) had a positive reaction. In CholU patients, we saw a highly significant correlation of sensitization to supMGL_1304 with wheal size in the ASwST (r_s_ = 0.7, *P* = 0.002, n = 19). Patients with IgE against supMGL_1304 had significantly larger wheals in the ASwST compared with the rest of the patients (mean 10.5 ± 2.7 mm vs. 5.0 ± 4.0 mm, *P* = 0.026; Fig. 2). No correlation or differences were seen with the wheal size of the histamine positive control (data not shown).

Serum autoreactivity and Ach i.c. reactivity were also assessed in both CholU patients and healthy controls. Twelve (31.6%) of the CholU patients but only 7 (10.1%) of the healthy controls showed a positive reaction in the ASST (*P* = 0.008). In contrast to the ASwST, no significant correlation with the sensitization to supMGL_1304 was seen (r_s_ = −0.44, *P* = 0.06, n = 19). None of the patients with elevated IgE levels to supMGL_1304 had a positive reaction in the ASST and only one of these patients developed a wheal in the ACh i.c test. However, all patients with elevated IgE levels to supMGL_1304 developed a flare reaction in the ACh i.c. test, compared to the remaining patients where only about 50% exhibited such a skin reaction (Table 2).

**Table 2:** Clinical characteristics of CholU patients with elevated specific IgE levels against supMGL-1304 (MG+) and CholU patients with normal low supMGL-1304 (MG−)

	MG+ n = 4	MG− n = 21	*P* value
Age [years]			
Median (range)	35.5 (21–57)	31.0 (18–55)	
Mean ± SD	37.3 ± 9.0	32.9 ± 2.5	0.504
Sex			
M:F	0:4	11:10	0.105
BMI [kg/m^2^]			
Median (range)	21.4 (20.2–25.2)	24.7 (20.0–32.3)	
Mean ± SD	22.1 ± 1.1	25.1 ± 0.8	0.119
Clinical data			
Persistence of disease [years]			
Median (range)	3.5 (1.0–29.0)	5.0 (1.0–20.0)	
Mean ± SD	9.3 ± 6.6	7.6 ± 1.3	0.681
CholUSI		n = 19	
Median (range)	16.5 (15–19)	16.0 (10–20)	
Mean ± SD	16.7 ± 0.8	16.0 ± 0.5	0.540
UAS Provo	**n = 3**	**n = 10**	
Median (range)	**5 (5, 6)**	**4 (3–5)**	
Mean ± SD	**5.3 ± 0.5**	**4.1 ± 0.7**	**0.036**
Itch Score Provo	*n *=* 3*	*n *=* 10*	
Median (range)	*2 (2, 3)*	*1 (1, 2)*	
Mean ± SD	*2.3 *±* 0.5*	*1.3 *±* 0.5*	*0.069*
Wheal Score Provo	n = 3	n = 10	
Median (range)	3 (3)	3 (2, 3)	
Mean ± SD	3 ± 0	2.8 ± 0.4	0.167
Atopic features			
Erlangen Atopy score		**n = 20**	
Median (range),	**14.5 (12–20)**	**9.0 (5–14)**	
Mean ± SD	**15.3 ± 1.7**	**9.0 ± 0.7**	**0.026**
Total IgE [kU/l]		n = 15	
Median (range)	1360.5 (278.0–4377.0)	71.9 (14.8–600.0)	
Mean ± SD	1844.0 ± 886.0	154.5 ± 169.2	0.152
Pos. Sx1 screening (inhalative allergen mix)	n = 3	n = 15	
	2 (67%)	9 (60%)	1.0
Pos. sIgE to other skin resident fungi		n = 17	
	**3 (75%)**	**1 (6.3%)**	**0.012**
Skin testings			
ASwST 1:100 pos. (n)	4 (100%)	15 (71%)	0.540
ASwST wheal size diameter (mm)		**n = 20**	
Median (range)	**9.5 (0.8–1.5)**	**5.5 (0–1.3)**	
Mean ± SD	**10.5 ± 2.7**	**5.6 ± 4.2**	**0.04**
ASwST flare size diameter (mm)		*n *=* 20*	
Median (range)	*33.5 (1.4*–*3.7)*	*12.0 (0*–*4.5)*	
Mean ± SD	*29.5 *±* 0.9*	*17 *±* 4*	*0.060*
ASST pos (n)	0 (0%)	5 (24%)	0.55
ASST wheal size diameter (mm)		**n = 20**	
Median (range)	**0 (0)**	**0 (0–9)**	
Mean ± SD	**0 ± 0**	**1.9 ± 3.2**	**0.017**
ASST flare size diameter (mm)		*n *=* 20*	
Median (range)	*0 (0*–*4)*	*0 (0*–*19)*	
Mean ± SD	*10 *±* 1.7*	*5.2 *±* 8.9*	*0.075*
ACh i.c. pos (n)	1 (25%)	10 (47.6%)	0.604
ACh i.c wheal size diameter (mm)			
Median (range)	0 (0–0.8)	0 (0–10)	
Mean ± SD	2.0 ± 1.2	3.1 ± 3.9	0.626
ACh i.c. flare size diameter (mm)			
Median (range)	5.0 (5–55)	5.0 (0–35)	
Mean ± SD	19 ± 20.9	13.3 ± 1.6	0.0675

Statistical significant differences are shown in bold, trends in italics

*ACh i.c.* acetylcholine intracutaneous injection, *ASwST* autologous sweat skin test, *ASST* autologous serum skin test, *BMI* body mass index, *CholUSI* Cholinergic Urticaria Severity Index, *Provo* provocation, *SD* standard deviation, *UAS* urticaria activity score

### Sensitization to supMGL_1304 was associated with atopic predisposition (Erlangen Atopy score, total IgE)

In CholU patients, levels of IgE against supMGL_1304 showed a positive correlation with the Erlangen atopy score (r_s_ = 0.53, *P* = 0.02, n = 19) and with total IgE serum levels (r_s_ = 0.54, *P* = 0.04, n = 19), but not with sensitization to inhalant allergens (SX1 r_s_ = 0.31, *P* = 0.30, n = 13).

### Sensitization to supMGL_1304 was associated with female sex and higher UASprovo scores, driven by intensity of itch

All four patients with clearly elevated serum levels of IgE anti supMGL_1304 were females. Due to the low numbers, interpretation of the statistical significance of these results must be made with caution.

Clinically, levels of IgE against supMGL_1304 were significantly correlated to the skin reactivity in the pulse controlled ergonometry test. There was a good and significant correlation with the UASprovo sum score (r_s_ = 0.84, *P* = 0.004, n = 10). Analysis of the wheal and itch scores revealed that this correlation was driven by itch (r_s_ = 0.84, *P* = 0.006, n = 10), but not wheal (r_s_ = 0.41, *P* = 0.40, n = 10), indicating that sensitization to supMGL_1304 was associated with more severe itch in the CholU patients.

No correlation was seen with age, disease persistance, frequency of symptoms or factors that trigger sweating (data not shown). Specific IgE levels against supMGL_1304 in CholU patients were negatively correlated with body weight (r_s_ = − 0.55, *P* = 0.02, n = 19), indicating that sensitization to supMGL_1304 is not associated with large skin surface area/obesity.

## Discussion

To our knowledge, this is the first screening of CholU patients for specific IgE against skin fungi in European CholU patients. Sensitization to *Malassezia globosa* is well documented in Japanese patients [17].

We found that a small subgroup of patients (up to 26%) have IgE against *Candida albicans*, *Malassezia furfur* and *Trichophyton mentagrophytes*. Interestingly, there was a high correlation between sensitization to the different fungi, indicating that there is a subgroup of CholU patients who are prone to sensitization against skin resident fungi. In patients with atopic dermatitis, skin resident fungi are thought to play an important role in the pathogensis of the disease via altered cytokine responses to the fungus [29, 30]. Whether similar responses are induced in CholU has not yet been investigated.

The proportion of patients with elevated serum levels of IgE against *Malassezia globosa* was considerably lower in our study than in a previous study of Japanese patients [17] (12% vs. 58%). This may be due to the difference in climate between Germany and Japan, which could lead to lower levels of *Malassezia globosa* colonization in German patients [31, 32]. Alternatively, there may be genetic differences between the German Caucasian and Japanese populations that affect sensitivity to skin resident fungi.

In our study, patients with sensitization to supMGL_1304 had more pronounced skin reactions in the ASwST and a correlation of the wheal size in the ASwST with levels of IgE against supMGL_1304. Several mechanisms could explain this observation. Firstly, the pronounced skin reaction may be due to sensitization to *Malassezia globosa* and a true IgE-mediated Type I allergy to *Malassezia globosa.* This may be facilitated by sweat, components of which have been shown to increase the growth rate of *Malassezia globosa* [32]. Alternatively, sensitization to *Malassezia* could have a direct action on mast cells, increasing histamine content and interleukin-6 production [33], and thereby augmenting histamine release caused by other activation mechanisms. A combination of these two mechanisms is also possible. To answer these questions, functional test using basophils or mast cells would be needed, and should be performed in the future.

The more pronounced skin reactions in the ASwST on the other hand cannot be explained with a general hyperreactive skin in the *supMGL_1304* sensibilized patients, as their reactivity to histamine per se, did not show any detectable differences. Also no correlation with the ASST has been seen.

Overall, there was a high number of patients, who showed positive reactions in the ASwSt, but had no detectable sensitizations to the analyzed fungi. This may be due to sensitization to yet unidentified sweat antigens, directly mast cell-activating factors, or histamine or similar mediators that are contained in the sweat of patients with CholU.

Sensitization to supMGL_1304 was strongly associated with atopic skin features and total IgE levels, but not with sensitization to common airborne/inhalant allergens. This is consistent with previous studies that showed a high rate of atopy in CholU patients [12, 34] and sensitization to supMGL_1304 in both CholU and atopic dermatitis patients [35]. This association fits the hypothesis that defects in the skin barrier could facilitate antigen uptake and subsequent sensitization [36]. Also, the microbes themselves could induce skin barrier defects that in turn initiate the sensitization [37], leading to a reinforcing feed-back loop.

Clinically, sensitization to supMGL_1304 was associated with higher symptom scores in the pulse-controlled ergonometry test, specifically itch. The reason for this remains unclear and warrants further investigation. To our knowledge, no other publication has reported such a correlation.

In our small cohort of four patients with elevated IgE levels to supMGL_1304, all patients were female. This was unexpected, as greater skin colonization by *Malassezia globosa* has been reported in males [31]. However, atopy has been associated with female sex in CholU patients [12], and we have shown a strong correlation between IgE against supMGL_1304 and atopy in our study. Overall, the number of patients sensibilised to supMGL_1304 was low and significant conclusions cannot be drawn. No correlation was seen with other assessed clinical factors such as age, disease persistance, frequency of symptoms or factors that trigger sweating.

The detection of specific IgE to *Malassezia globosa* raises the question of whether antimycotic treatment could be beneficial in CholU patients. In atopic dermatitis, clinical studies have not provided any clear evidence that antimycotic treatment leads to a significant improvement in symptoms [38, 39], but patients were usually not screened for sensitization to *Malassezia globosa* In CholU, desensitization therapy using autologous sweat or *Malassezia globosa* peptides has been shown to be beneficial for patients with intractable CholU due to sweat allergy [35, 40, 41]. Furthermore, intensified showering or tannin acid have been proposed as therapeutic regimens in patients with atopic dermatitis sensitized to *Malassezia globosa* [41]. Further work, including placebo-controlled studes, is needed in this area.

The main limitation of our study is the low number of CholU patients who displayed a sensitization to *Malassezia globosa* Furthermore, data on the actual skin residency of skin fungi, their subspecies and numbers were not captured.

## Conclusion

In summary, this study showed that a subgroup of German Caucasian CholU patients displayed sensitization to skin resident fungi and that this sensitization was associated with atopic predisposition and more pronounced skin reactions to sweat provocation tests. Anti-fungal treatments could be a therapeutic option in these patients, if skin colonization with fungi is present and increased.

## Data Availability

Raw data were generated at Charité–Universitätsmedizin Berlin, Germany. Derived data supporting the findings of this study are available from the corresponding author on request.

## Abbreviations

ACh: Acetylcholine
ASST: Autologous serum skin test
ASwST: Autologous sweat skin test
AU: Arbitrary unit
CholU: Cholinergic urticaria
i.c.: Intracutaneous
